# Musicality and social cognition in dementia: clinical and anatomical associations

**DOI:** 10.1093/braincomms/fcae429

**Published:** 2024-12-13

**Authors:** Jochum J van ‘t Hooft, Willem L Hartog, Michelle Braun, Dewi Boessen, Jay L P Fieldhouse, Marie-Paule E van Engelen, Ellen H Singleton, Artur C Jaschke, Rebecca S Schaefer, Vikram Venkatraghavan, Frederik Barkhof, Argonde C van Harten, Flora H Duits, Sigfried N T M Schouws, Mardien L Oudega, Jason D Warren, Betty M Tijms, Yolande A L Pijnenburg

**Affiliations:** Department of Neurology, Alzheimer Center Amsterdam, Vrije Universiteit Amsterdam, Amsterdam UMC Location VUmc, 1081 HZ Amsterdam, The Netherlands; Amsterdam Neuroscience, Neurodegeneration, 1081 HV Amsterdam, The Netherlands; Department of Neurology, Alzheimer Center Amsterdam, Vrije Universiteit Amsterdam, Amsterdam UMC Location VUmc, 1081 HZ Amsterdam, The Netherlands; Amsterdam Neuroscience, Neurodegeneration, 1081 HV Amsterdam, The Netherlands; Department of Neurology, Alzheimer Center Amsterdam, Vrije Universiteit Amsterdam, Amsterdam UMC Location VUmc, 1081 HZ Amsterdam, The Netherlands; Amsterdam Neuroscience, Neurodegeneration, 1081 HV Amsterdam, The Netherlands; Department of Neurology, Alzheimer Center Amsterdam, Vrije Universiteit Amsterdam, Amsterdam UMC Location VUmc, 1081 HZ Amsterdam, The Netherlands; Amsterdam Neuroscience, Neurodegeneration, 1081 HV Amsterdam, The Netherlands; Department of Neurology, Alzheimer Center Amsterdam, Vrije Universiteit Amsterdam, Amsterdam UMC Location VUmc, 1081 HZ Amsterdam, The Netherlands; Amsterdam Neuroscience, Neurodegeneration, 1081 HV Amsterdam, The Netherlands; Department of Neurology, Alzheimer Center Amsterdam, Vrije Universiteit Amsterdam, Amsterdam UMC Location VUmc, 1081 HZ Amsterdam, The Netherlands; Amsterdam Neuroscience, Neurodegeneration, 1081 HV Amsterdam, The Netherlands; Department of Neurology, Alzheimer Center Amsterdam, Vrije Universiteit Amsterdam, Amsterdam UMC Location VUmc, 1081 HZ Amsterdam, The Netherlands; Amsterdam Neuroscience, Neurodegeneration, 1081 HV Amsterdam, The Netherlands; Music Therapy, ArtEZ University of the Arts, 7511 PN Enschede, The Netherlands; Department of Psychiatry, University of Cambridge, Cambridge, UK; Department of Neonatology, University Medical Center Groningen, 9713 GZ Groningen, The Netherlands; Cambridge Institute for Music Therapy Research, Cambridge, UK; Health, Medical and Neuropsychology Unit, Institute of Psychology, Leiden University, 2333 AK Leiden, The Netherlands; Academy for Creative and Performing Arts, Leiden University, 2311 GZ Leiden, The Netherlands; Department of Neurology, Alzheimer Center Amsterdam, Vrije Universiteit Amsterdam, Amsterdam UMC Location VUmc, 1081 HZ Amsterdam, The Netherlands; Amsterdam Neuroscience, Neurodegeneration, 1081 HV Amsterdam, The Netherlands; Department of Radiology and Nuclear Medicine, Vrije Universiteit Amsterdam, Amsterdam UMC, 1081 HV Amsterdam, The Netherlands; UCL Institutes of Neurology and Healthcare Engineering, University College London, UK; Department of Neurology, Alzheimer Center Amsterdam, Vrije Universiteit Amsterdam, Amsterdam UMC Location VUmc, 1081 HZ Amsterdam, The Netherlands; Amsterdam Neuroscience, Neurodegeneration, 1081 HV Amsterdam, The Netherlands; Department of Neurology, Alzheimer Center Amsterdam, Vrije Universiteit Amsterdam, Amsterdam UMC Location VUmc, 1081 HZ Amsterdam, The Netherlands; Amsterdam Neuroscience, Neurodegeneration, 1081 HV Amsterdam, The Netherlands; Neurochemistry Lab, Department of Laboratory Medicine, Amsterdam UMC Location VUmc, 1081 HV Amsterdam, The Netherlands; Department of Psychiatry, Amsterdam UMC Location Vrije Universiteit Amsterdam, 1081 HV Amsterdam, The Netherlands; GGZ, InGeest Specialized Mental Health Care, Old Age Psychiatry, 1081 JC Amsterdam, The Netherlands; Department of Psychiatry, Amsterdam UMC Location Vrije Universiteit Amsterdam, 1081 HV Amsterdam, The Netherlands; GGZ, InGeest Specialized Mental Health Care, Old Age Psychiatry, 1081 JC Amsterdam, The Netherlands; Amsterdam Neuroscience, Mood, Anxiety, Psychosis, Sleep and Stress Program, 1081 HV Amsterdam, The Netherlands; Dementia Research Centre, UCL Queen Square Institute of Neurology, University College, London, UK; Department of Neurology, Alzheimer Center Amsterdam, Vrije Universiteit Amsterdam, Amsterdam UMC Location VUmc, 1081 HZ Amsterdam, The Netherlands; Amsterdam Neuroscience, Neurodegeneration, 1081 HV Amsterdam, The Netherlands; Department of Neurology, Alzheimer Center Amsterdam, Vrije Universiteit Amsterdam, Amsterdam UMC Location VUmc, 1081 HZ Amsterdam, The Netherlands; Amsterdam Neuroscience, Neurodegeneration, 1081 HV Amsterdam, The Netherlands

**Keywords:** music cognition, frontotemporal dementia, amusia, emotion recognition, theory of mind

## Abstract

Human musicality might have co-evolved with social cognition abilities, but common neuroanatomical substrates remain largely unclear. In behavioural variant frontotemporal dementia, social cognitive abilities are profoundly impaired, whereas these are typically spared in Alzheimer’s disease. If musicality indeed shares a neuroanatomical basis with social cognition, it could be hypothesized that clinical and neuroanatomical associations of musicality and social cognition should differ between these causes of dementia. We recruited 73 participants from the Amsterdam Dementia Cohort (*n* = 30 female; aged 50–78), of whom 23 had behavioural variant frontotemporal dementia, 22 Alzheimer’s disease and 28 were healthy controls. Musicality was assessed using a music–emotion recognition test, melody, tempo, accent and tuning subscores, a musicality summed score, the identification of auditory hedonic phenotypes and music emotion induction using skin conductance responses. Social cognition was assessed across multiple levels, including emotion recognition, theory of mind, socio-emotional sensitivity and understanding of social norms. We used ANCOVA to investigate subgroup differences in musicality and social cognition and linear regressions to investigate associations between musicality and social cognition. All analyses were adjusted for age, sex, musical training and mini mental state examination. Finally, we performed voxel-based morphometry analyses on T_1_-weighted MRI to study whether regions for musicality and social cognition overlapped anatomically. We found that patients with behavioural variant frontotemporal dementia performed worse on music–emotion recognition (all *P* < 0.001) and tempo recognition (all *P* < 0.05) compared with Alzheimer’s disease and on musicality summed score (all *P* = 0.02) compared to controls only. Furthermore, patients with behavioural variant frontotemporal dementia had lower mean skin conductance responses during emotion-inducing music, compared to Alzheimer’s disease (all *P* < 0.045). Worse music emotion recognition scores were associated with worse facial emotion recognition (*P* < 0.0001), worse theory of mind (*P* = 0.0005) and worse understanding of social norms (*P* = 0.01). Melody and tempo recognition were associated with facial emotion recognition and theory of mind, and accent recognition was associated with the theory of mind. Music emotion recognition and tempo recognition were also associated with executive functions. Worse music emotion recognition, melody recognition, tempo recognition, facial emotion recognition and theory of mind scores were all related to atrophy in the anterior temporal regions and the fusiform gyri, which play a role in multisensory integration, and worse tempo recognition was associated with atrophy of the anterior cingulate cortex. These results support the idea that musicality and social cognition may share a neurobiological basis, which may be vulnerable in behavioural variant frontotemporal dementia.

## Teaser

In the current study, van ‘t Hooft *et al*. report that musicality might be affected in behavioural variant frontotemporal dementia but not in Alzheimer’s disease and that musicality might be associated with emotion recognition, theory of mind and with brain regions that support social cognition.

## Graphical Abstract

**Graphical Abstract:**
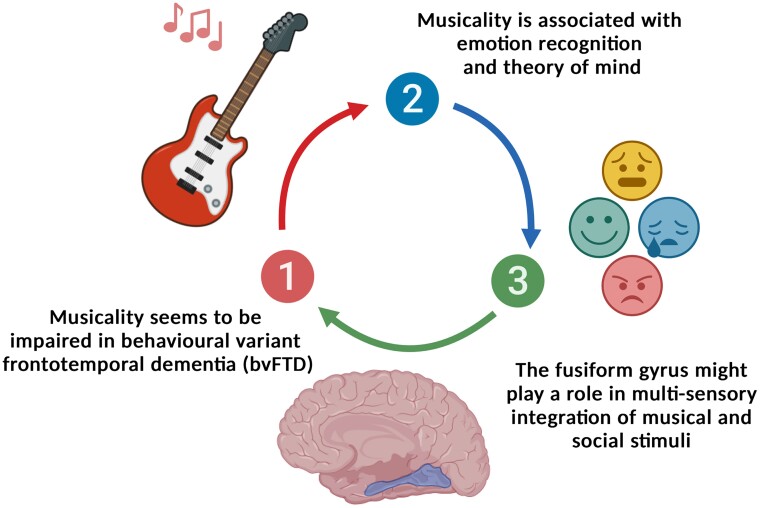

## Introduction

Frontotemporal dementia is characterized by profound impairment in social cognition. A problem is, however, that currently there is no standard for measuring social cognition, which hampers the diagnostic process. Moreover, for early diagnosis, it is important to identify metrics that may be more sensitive to the earliest changes in social cognition. Musicality has been suggested as a probe for social cognition abilities.^1-3^ Musicality refers to both productive and receptive musical abilities such as musical understanding, appreciation, as well as a sense of pitch, rhythm and emotional value.^4,5^ Social interactions in daily life depend in large part on decoding acoustic signals, and musical capacities might therefore be useful in a social context. This hypothesis is supported by clinical and evolutionary studies^2,3,6^ that have suggested overlapping properties of musicality and social cognition.

We and other groups previously found that patients with behavioural variant frontotemporal dementia (bvFTD) exhibited notable impairments in music emotion recognition abilities^7-9^ and inferring mental states from musical pieces (i.e. ‘musical mentalizing’).^1^ Furthermore, hedonic musical changes such as musicophilia (abnormal craving for music), musical anhedonia (strongly decreased interest in music) and change in musical taste are often present in bvFTD patients.^10-13^ Such changes were found to be associated with more atrophy in cortical areas that are related to the salience network and the semantic appraisal network^1,7,14^ and for hedonic changes with more atrophy in temporo-insulo-striatal regions.^10-13^ Those brain areas have also been found to play a role in social cognition,^15^ suggesting that musicality and social cognition share a neuroanatomical basis. We previously identified that the salience network and semantic appraisal network are involved in both music processing and social cognition and largely overlap with atrophy patterns in bvFTD.^16^ From this literature, it can be hypothesized that if musicality and social cognition share neuroanatomical basis, then typical socio-emotional changes in bvFTD might be accompanied by changes in music processing, while no alterations in musicality would be expected in typical Alzheimer’s disease,^17^ given the spared social cognitive abilities in this disorder.

In the current study, we investigated this hypothesis in participants with bvFTD, Alzheimer’s disease and cognitively healthy controls. We studied how musicality is related to social cognition, and if these capacities were related to atrophy in specific cortical areas as measured on MRI.

## Materials and methods

### Participants

Participants were recruited prospectively and consecutively through the Amsterdam Dementia Cohort^18^ from the tertiary outpatient memory clinic of the Alzheimer Center Amsterdam. Patients were diagnosed with bvFTD or Alzheimer’s disease according to current consensus criteria.^19,20^ As controls, we used subjects who presented with subjective cognitive decline when they had negative amyloid biomarkers, and no cognitive dysfunction was found following the extensive diagnostic procedure of the Amsterdam Dementia Cohort.^18^ Patients with Alzheimer’s disease were required to have abnormal amyloid biomarkers (in CSF or on PET), and bvFTD patients were required to have a supportive frontal fluorodeoxyglucose positron emission tomography pattern of hypometabolism or atrophy pattern on MRI. Participants were excluded from the present study if they were unable to perform the procedures or give informed consent (due to language or cognitive impairments), had hearing problems or used hearing aids. All participants underwent a standardized neuropsychological test battery that tested the following cognitive domains: memory, attention, language and executive functioning. Memory was assessed with the Visual Association Test and the Rey Verbal Auditory Memory Test immediate and delayed recall scores; Attention was assessed with the Trail Making Test A, and Stroop word and colour subtest; language was assessed with the Visual Association Test naming condition and Animal Fluency and executive functioning was assessed with the Trail Making Test B, Stroop colour-word subtest and the Letter Fluency task. Domain *z*-scores were referenced to a group of 533 healthy amyloid-negative subjects^21^ of the Amsterdam Dementia Cohort.

#### Ethical approval and consent to participate

The ethics committees of the Amsterdam UMC Location VUmc provided approval for the conduction of this work (submission 2020.0602), and all patients gave informed consent to participate in the research, and for the publication of results based on their data. All participants signed informed consent in accordance with the declaration of Helsinki.

### Musicality tasks

The following domains of musicality were investigated: music emotion recognition, general musicality (with melody, tuning, accent, tempo and summed subscores), music emotion induction using skin conductance responses (SCRs) and auditory hedonic phenotypes.

#### Music emotion recognition

Music emotion recognition was assessed using the musical emotion discrimination task (MEDT).^22^ The MEDT consists of 18 audio clips, each with two excerpts of the same melody that differ only in the emotional expression conveyed through the performance (happy, sad, angry or tender). Participants were instructed to indicate which version they felt was most representative of a given emotion (e.g. ‘listen to the following clips and select which one sounds angrier to you. Select 1 for the clip heard before the beep, or 2 for the clip heard after the beep’).

#### General musicality

General musicality was assessed with the mini version of the profile of music perception skills (Mini-PROMS), which consists of 4 subtests: melody, tuning, accent and tempo.^23^ Participants listened to two reference stimuli followed by a probe stimulus and judged whether the probe stimulus was the same or different in the specific modality (e.g. melody, tempo, accent and tuning) compared to the reference stimulus. The PROMS melody stimuli consisted of 8 monophonic notes of a constant rhythm; the PROMS tempo consisted of musical excerpts having either the same or different tempos; the PROMS accent consisted of stimuli with different emphasis in rhythmic patterns and the PROMS tuning stimuli consisted of a C chord on a piano where the E note was shifted out of its proper frequency (shifts from 10 to 50 cents).^24^

#### Music emotion induction

Biometric responses to emotional music were assessed with SCRs. Participants listened to 6 unfamiliar music excerpts of three basic emotions (2 excerpts per emotion): happy, fearful and sad^25^ (see Supplementary Table 1 for the list of music excerpts). During the music pieces, SCR was measured using a Shimmer3 GSR+ sensor as a proxy for physiological arousal. Mean SCR amplitude in MicroSiemens (µS) were collected every 1000 ms, which were averaged to calculate a mean SCR during each musical emotion. The mean SCR of 1 s after the start of the stimulus until 1 s after the stimulus ended were selected to take into account the delay of SCR to physiological responses. One minute of rest preceded the music pieces to neutralize the SCR of participants.

#### Auditory hedonic phenotypes and musical training

In line with prior studies,^9,12,13^ we used an informant-based questionnaire to assess the auditory hedonic phenotypes musicophilia, musical anhedonia, change in musical taste, music aversion and sound aversion. Musicophilia was defined as compulsive music listening of at least 10 h per week with a definite increase compared with premorbid levels, and musical anhedonia was defined as a definite decrease in music interest compared with premorbid levels. The musical training subscore of the Goldsmiths Musical Sophistication Index was used to assess musical training.^5^

### Social cognition

Three components of social cognition were assessed^26^: perception of social information (e.g. emotion recognition), interpretation of social information (e.g. theory of mind) and social reasoning and social behaviour.

#### Perception of social information

The Ekman-60-faces test assessed facial emotion recognition, within the perception domain of social cognition.^27^ Participants viewed 60 photos of faces with emotional expressions and judged which of the six basic emotions were displayed: happiness, sadness, anger, surprise, disgust and fear.

#### Interpretation of social information

The hinting task and the revised self-monitoring scale (RSMS) assessed the awareness and sensitivity to socio-emotional cues and behaviour, within the interpretation domain of social cognition. The hinting task assesses the ability to recognize the intention of indirect speech (i.e. cognitive theory of mind). Participants listened to 10 short passages of an interaction between two characters. Each passage ends with one character dropping a social hint, and participants judged what the character truly meant (e.g. ‘It is almost Rebecca’s birthday, she tells her father ‘I love animals, especially dogs’. What is Rebecca trying to convey with this message?’). Participants received 2 points for a correct answer, if a response is inaccurate, a second hint was delivered for partial credit.^28,29^ The RSMS is a 13-item informant-based questionnaire that assesses socio-emotional sensitivity. It consists of a total score and two subscores: modifying self-presentation (RSMS-SP) and sensitivity to expressive behaviour of others (RSMS-EX).^30^

#### Social reasoning and behaviour

The Dutch version of the Social Norms Questionnaire (SNQ) assessed the understanding of social norms and social boundaries within the regulation and reasoning domain of social cognition.^31^ A total score and two error scores can be derived from the SNQ: (i) the total knowledge of social norms; (ii) the tendency to break social norms and (iii) the tendency to overadhere to social norms (i.e. apply social norms too rigidly).

### Experimental procedure

Both the musicality and social cognition tests were administered using a laboratory setting on a computer with iMotions software (iMotions version 8.0; https://imotions.com/). The order of tests within the test battery were identical for each participant, but the stimuli within tests were randomized. Participants were connected to the SCR sensor on the non-dominant index finger and ring finger. Participants were provided with wired over-ear headphones (Devine PRO 4000) during the experiments, and sound calibration set to patient comfort level at the beginning of the musicality tests.

### Statistical analysis

Statistical analyses were performed with R (version 4.1.0). For demographical characteristics and group comparisons, *t*-test, Kruskal–Wallis test and χ2 test were used where appropriate. Group differences on musicality test scores (MEDT, PROMS total score and subscores) and the SCR were analysed using ANCOVA’s, and emmeans for *post hoc* comparison of group means adjusted for age, sex, musical training and mini mental state examination (MMSE). Next, we tested the discriminatory ability of each musicality and social cognition test, by building logistic regression model that tested the ability of each musicality and social cognition test (predictor) adjusted for age, sex and education to discriminate bvFTD from Alzheimer’s disease and healthy controls combined (outcome). We created receiver operating characteristic (ROC) curves and calculated the area under the curve (AUC) using the pROC package in R.^32^ Next, for each musicality test (predictor), we assessed the relationship with each social cognition test score (outcome) with linear regression models, adjusted for age, sex, musical training and MMSE. We repeated these analyses stratified on diagnostic groups. We also studied if music was related to general executive functioning as a non-social control condition. A threshold of *P* < 0.05 was accepted as a statistically significant difference or predictive value in all comparisons and regression models.

### Brain MRI acquisition, pre-processing and VBM analysis

We studied anatomical associations of the musicality tests (MEDT, PROMS melody, PROMS tempo, PROMS accent and PROMS tuning) and social cognition (Ekman-60-faces test, the hinting task and SNQ) using structural MRI. Briefly, brain MRI scans were all acquired at the Amsterdam UMC, location VUmc on either a Philips Ingenuity, Siemens MAGNETOM Vida, General Electric Discovery MR750 or a Vantage Titan scanner. All scanners had a field strength of 3 T with voxel dimensions ranging from 0.9 to 1.2 mm. The acquired brain images were visually assessed to exclude images containing artefacts. Pre-processing of the MR images was performed using the DARTEL toolbox of SPM12 (www.fil.ion.ucl.ac.uk/spm) running under MATLAB 9.13. Normalization, segmentation, modulation and smoothing of grey and white matter images were done using the default parameter settings. The final template brain image was created by warping the native space brain images to the final DARTEL template in Montreal Neurological Institute stereotactic space, as well as calculating the average of the brain images. Adjusting for individual differences in head size was done by calculating the total intracranial volume by summing grey and white matter, and cerebrospinal fluid volumes after segmentation of these tissue classes for each patient. A linear multiple regression design in SPM12 was used to investigate the voxel-wise associations of performance on the musicality and social cognition tasks (predictor) on grey matter volume. Age, sex, total intracranial volume, diagnostic subgroup and scanner type were included as covariates of no interest. An automatic explicit mask was applied after model estimation. Statistical parametric maps of grey matter volume correlating with the performance on the musicality tasks were examined at an uncorrected threshold of *P* < 0.001, with clusters >50 voxels. Overlapping brain regions of the significant associations in the regression analyses were investigated using Mango software (Version 4.1).

## Results

### Clinical and behavioural characteristics

The demographic and clinical characteristics can be found in Table 1. A total of 73 participants were included of which 23 patients with bvFTD (22% female), 22 patients with Alzheimer’s disease (33% female) and 28 controls (64% female; Table 1). There were more male participants in the bvFTD group compared with the healthy participants group (*P* = 0.01). There were no statistical differences in age, education, handedness and musical training between the participants, and no differences in MMSE and symptom duration between bvFTD and Alzheimer’s disease patient groups. Musical training ranged widely across the cohort (mean ± SD = 16.9 ± 8.1, range = 7–41) and was similar to previous clinical samples in older adults.^33^ The musicality tests were discontinued in 4 bvFTD patients due to excessive cognitive and behavioural problems. Musicophilia was present in 6 bvFTD participants (26%), change in musical taste in 7 bvFTD patients (30.4%), musical anhedonia in 3 bvFTD patients (13%), 1 Alzheimer’s disease patient (4.5%), and 2 healthy controls (7.1%), music aversion in 2 bvFTD patients (8.7%) and 3 healthy controls (10.7%), and sound aversion was present in 4 bvFTD patients (17.4%), 4 Alzheimer’s disease patients (18.2%) and 6 healthy controls (21.4%). Forty-eight subjects (12 bvFTD, 14 Alzheimer’s disease, 22 healthy controls) had volumetric MRI data available. The SCR data of 1 patient with bvFTD and 1 healthy control subject were excluded due to technical failure.

**Table 1:** Demographic characteristics

	bvFTD	Alzheimer’s disease	Controls	*P*-value
Demographic				
No. (M:F)	23 (18:5)	22 (15:7)	28 (10:18)	bvFTD versus controls, *P* = 0.01**
Age (years)	65.4 (6.2)	65.5 (6.4)	64.4 (6.2)	0.78
Education (years)	11.9 (2.8)	12.8 (3.3)	13.3 (2.5)	0.15
Handedness (R:L)	21:2	22:0	24:4	0.19
MMSE	26.0 (1.8)	26.1 (2.7)	29.4 (0.9)	bvFTD < controls, *P* < 0.0001*** bvFTD = Alzheimer’s disease, *P* = 0.8 Alzheimer’s disease < controls, *P* < 0.0001**
Genetic mutations	7 (4 c9orf72, 2 MAPT, 1 TARDBP)	0	n.a.	n.a.
Symptom duration (years)	3.9 (2.2)	3.8 (2.3)	n.a.	n.a.
Musical training^a^ (7–49)	16.3 (8.2)	16.4 (8.5)	17.7 (8.0)	0.79
Musicophilia (%)	6 (26.1%)	0 (0%)	0 (0%)	n.a.
Change in musical taste (%)	7 (30.4%)	0 (0%)	0 (0%)	n.a.
Musical anhedonia (%)	3 (13.0%)	1 (4.5%)	2 (7.1%)	n.a.
Music aversion (%)	2 (8.7%)	0 (0%)	3 (10.7%)	n.a.
Sound aversion (%)	4 (17.4%)	4 (18.2%)	6 (21.4%)	n.a.
MRI scan available (%)	12 (52.2%)	14 (63.6%)	22 (78.6%)	n.a.
Neuropsychological				
Attention domain *Z*-score	−0.75 (1.8)	−0.62 (0.8)	0.54 (0.7)	bvFTD < controls, *P* = 0.0013** bvFTD = Alzheimer’s disease, *P* = 0.74 Alzheimer’s disease < controls, *P* = 0.0029**
Executive domain *Z*-score	−0.48 (1.31)	−0.95 (0.8)	0.51 (0.8)	bvFTD < controls, *P* = 0.018* bvFTD = Alzheimer’s disease, *P* = 0.20 Alzheimer’s disease < controls, *P* = 0.0002***
Memory domain *Z*-score	−1.39 (1.4)	−2.71 (1.7)	0.46 (0.7)	bvFTD < controls, *P* = 0.0001*** bvFTD > Alzheimer’s disease, *P* = 0.0046** Alzheimer’s disease < controls, *P* < 0.0001**
Language domain *Z*-score	−1.29 (1.2)	−0.42 (0.5)	0.24 (0.6)	bvFTD < controls, *P* < 0.0001*** bvFTD < Alzheimer’s disease, *P* = 0.005** Alzheimer’s disease < controls, *P* = 0.019*

Data are presented as *N*, mean (SD). *Z*-scores of each cognitive domain were calculated in reference to a cognitively normal amyloid-negative control group (*n* = 533; Groot *et al.*^21^). Cognition scores were adjusted for age and sex. MMSE, mini mental state examination; n.a., not applicable. ^a^The musical training subscale of the Goldsmiths Musical Sophistication Index was used. Significant associations are displayed in bold. *P ≤ 0.05; **P ≤ 0.01; ***P ≤ 0.001.

### Performance on the musicality tests, music emotion induction and ROC curves

**Figure 1:**
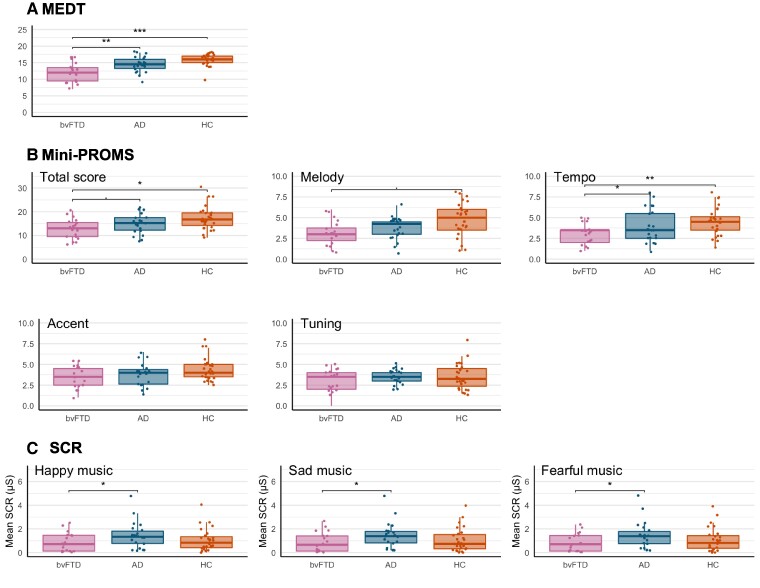
**Performance on the musicality tasks, SCRs.** (**A**) Performance on the MEDT. (**B**) Performance on the Mini-PROMS, total score and subscores (melody, tempo, accent and tuning). (**C**) SCRs to emotional music. The group differences were investigated using ANCOVA’s adjusted for age, sex, musical training and MMSE. *P* ≤ 0.1; *P ≤ 0.05; **P ≤ 0.01; ***P ≤ 0.001; MEDT, musical emotion discrimination task; Mini-PROMS, mini version of the profile of music perception skills; SCR, skin conductance response.

**Figure 2:**
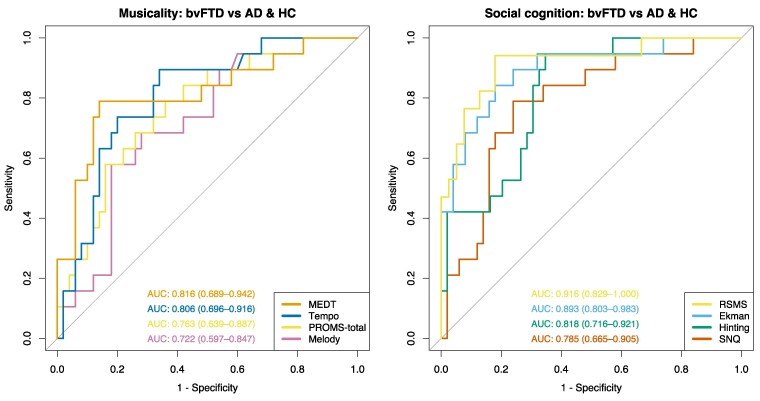
**ROC curves of the musicality and social cognition tests.** The ROC curves investigated the ability of each musicality and social cognition test (predictor) to discriminate bvFTD from Alzheimer’s disease and healthy controls combined (outcome) using logistic regression analysis adjusted for adjusted for age, sex and education using the pROC package in R.

The results of the musicality and social cognition tests, mean SCR during emotion-inducing music and ROC curves are plotted in Figs 1 and 2 are summarized in Table 2. The patients with bvFTD performed worse on the MEDT (*P* = 0.0002), PROMS total (*P* = 0.022), PROMS tempo (*P* = 0.0016) compared to healthy controls and worse on the MEDT (*P* = 0.0034) and PROMS tempo (*P* = 0.049) compared to patients with Alzheimer’s disease. There was a nonsignificant worse performance on the PROMS melody (*P* = 0.08) and PROMS total (*P* = 0.059) in bvFTD compared to Alzheimer’s disease. Patients with Alzheimer’s disease did not perform worse on any of the musicality tasks compared to controls. Patients with bvFTD showed lower mean SCR during the emotion-inducing music compared to Alzheimer’s disease (all *P* < 0.045). Next, we calculated the ability of the musicality and social cognition tests to discriminate bvFTD from Alzheimer’s disease and controls using ROC curves. First, we calculated the AUC of the musicality tests and found an AUC of 0.82 (95% CI: 0.69–0.94) for the MEDT scores, 0.81 (95% CI: 0.70–0.92) for tempo recognition scores, 0.72 (95% CI: 0.60–0.85) for melody recognition scores and 0.76 (95% CI: 0.64–0.89) for the PROMS total scores. For the social cognition tests, we found an AUC of 0.92 (95% CI: 0.83–1) for the RSMS, 0.89 (95% CI: 0.80–0.98) for the Ekman-60-faces test scores, 0.81 (95% CI: 0.72–0.92) for the hinting task and 0.79 (95% CI: 0.67–0.91) for the SNQ.

**Table 2:** Performance on the musicality tasks, social cognition tasks and skin conductance responses to emotional music

	bvFTD	Ad	Controls	*P*-value^a^
Musicality				
MEDT (max 18)	12.3 (2.9)	14.5 (2.4)	15.8 (1.7)	bvFTD < controls, *P* = 0.0002*** bvFTD < Ad, *P* = 0.0034** Ad = controls, *P* = 0.14
				
PROMS total score (max 36)	12.8 (4.2)	15.4 (4.3)	16.6 (4.9)	bvFTD < controls, *P* = 0.022* bvFTD < Ad, *P* = 0.059 Ad = controls, *P* = 0.46
PROMS Melody (max 10)	3.4 (1.4)	4.0 (1.4)	4.4 (2.0)	bvFTD < controls, *P* = 0.08 bvFTD = Ad, *P* = 0.2 Ad = controls, *P* = 0.51
PROMS Tempo (max 8)	2.7 (1.2)	3.7 (1.9)	4.7 (1.6)	bvFTD < controls, *P* = 0.0016** bvFTD < Ad, *P* = 0.049* Ad = controls, *P* = 0.11
PROMS Accent (max 10)	3.7 (1.3)	4.0 (1.4)	4.0 (1.3)	bvFTD = controls, *P* = 0.45 bvFTD = Ad, *P* = 0.38 Ad = controls, *P* = 0.99
PROMS Tuning (max 8)	3.0 (1.4)	3.6 (0.8)	3.4 (1.5)	bvFTD < controls, *P* = 0.35 bvFTD < Ad,*P* = 0.14 Ad = controls, *P* = 0.74
				
Social cognition				
Ekman-60-faces (max 60)	38.8 (8.0)	47.0 (4.4)	49.0 (4.8)	bvFTD < controls, *P* < 0.0001*** bvFTD < Ad, *P* < 0.0001*** Ad = controls, *P* = 0.26
Hinting task (max 20)	14.8 (3.8)	17.1 (1.8)	18.1 (1.3)	bvFTD < controls, *P* = 0.0002*** bvFTD < Ad, *P* = 0.0017** Ad = controls, *P* = 0.22
RSMS total (max 65)	26.7 (10.2)	42.3 (9.0)	41.8 (10.0)	bvFTD < controls, *P* = 0.0001*** bvFTD < Ad, *P* < 0.0001*** Ad = controls, *P* = 0.51
RSMS-EX (max 30)	9.0 (5.2)	18.1 (5.5)	20.0 (5.2)	bvFTD < controls, *P* < 0.0001*** bvFTD < Ad, *P* < 0.0001*** Ad = controls, *P* = 0.40
RSMS-SP (max 35)	17.0 (6.2)	18.5 (4.5)	23.2 (5.2)	bvFTD < controls, *P* = 0.019* bvFTD < Ad, *P* = 0.0007*** Ad = controls, *P* = 0.70
SNQ total (max 22)	16.4 (2.3)	18.5 (1.6)	18.4 (1.9)	bvFTD < controls, *P* = 0.06 bvFTD < Ad, *P* = 0.018* Ad = controls, *P* = 0.88
SNQ break score	2.0 (1.5)	1.1 (1.1)	1.7 (1.5)	bvFTD = controls, *P* = 0.57 bvFTD < Ad, *P* = 0.05 Ad = controls, *P* = 0.26
SNQ over adherence score	3.0 (2.1)	2.4 (1.6)	1.1 (1.2)	bvFTD < controls, *P* = 0.078 bvFTD = Ad, *P* = 0.22 Ad = controls, *P* = 0.44
Skin conductance responses^b^				
Happy music	0.73 (0.8)	1.30 (1.1)	1.19 (1.0)	bvFTD = controls, *P* = 0.33 bvFTD < Ad, *P* = 0.039* Ad = controls, *P* = 0.41
Sad music	0.74 (0.9)	1.32 (1.1)	1.18 (1.0)	bvFTD = controls, *P* = 0.37 bvFTD < Ad, *P* = 0.045* Ad = controls, *P* = 0.39
Fearful music	0.72 (0.7)	1.35 (1.1)	1.19 (1.0)	bvFTD = controls, *P* = 0.34 bvFTD < Ad, *P* = 0.031* Ad = controls, *P* = 0.35

Data are presented as mean (SD). MEDT, musical emotion discrimination task; Mini-PROMS, mini version of the profile of music perception skills; n.s., not significant; SCR, skin conductance response; SNQ, social norms questionnaire; RSMS, revised self-monitoring sale. ^a^All analyses were adjusted for age, sex, musical training and MMSE; ^b^Mean SCR amplitude is shown in MicroSiemens (µS). Significant associations are displayed in bold. *P ≤ 0.05; **P ≤ 0.01; ***P ≤ 0.001.

### Clinical associations of musicality and social cognition

**Figure 3:**
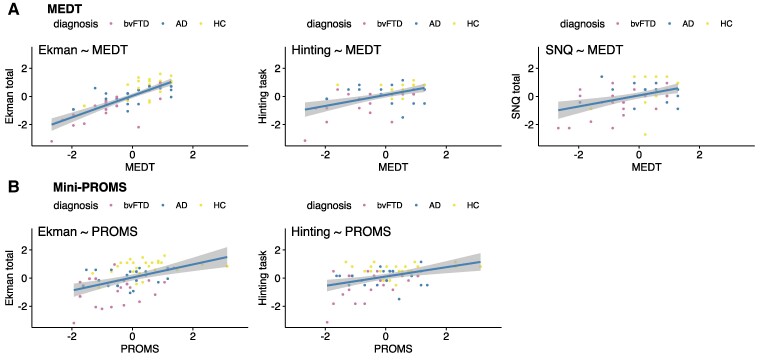
**Association between musicality and social cognition.** (**A**) Significant associations of performance on the MEDT and the social cognition tests. The MEDT was positively associated with performance on the Ekman-60-faces test (visual emotion recognition), the hinting task (theory of mind) and the SNQ (understanding social norms). (**B**) Significant associations of performance on the Mini-PROMS—total score. The PROMS—total score was positively associated with performance on the Ekman-60-faces test (visual emotion recognition) and the hinting task (theory of mind). All scores were *z*-transformed. The regression coefficients of the subtests of the Mini-PROMS can be found in Table 3. A linear regression model was used to investigate the association of each musicality test (predictor) and social cognition test score (outcome) adjusted for age, sex, musical training and MMSE. MEDT, musical emotion discrimination task; Mini-PROMS, mini version of the profile of music perception skills; Ekman, Ekman-60-faces test; Hinting, hinting task.

The associations of musicality and social cognition *z*-scores are plotted in Fig. 3, and regression coefficients are summarized in Table 3. Worse performance on the MEDT was associated with worse facial emotion recognition [*P* < 0.0001; *P*(FDR-corrected) < 0.0001], the hinting task [*P* = 0.00053; *P*(FDR-corrected) = 0.0024], the SNQ total score [*P* = 0.01; *P*(FDR-corrected) = 0.027] and general executive functioning [*P* = 0.027; *P*(FDR-corrected) = 0.049]. The PROMS total score was associated with the hinting task [*P* = 0.0005; *P*(FDR-corrected) = 0.0046] and facial emotion recognition [*P* = 0.002; *P*(FDR-corrected) = 0.01]; melody recognition was associated with facial emotion recognition [*P* = 0.0038; *P*(FDR-corrected) = 0.027] and the hinting task [*P* = 0.0059; *P*(FDR-corrected) = 0.027]; tempo recognition was associated with facial emotion recognition [*P* = 0.0066; *P*(FDR-corrected) = 0.03], the hinting task [*P* = 0.0069; *P*(FDR-corrected) = 0.03] and general executive functioning [*P* = 0.014; *P*(FDR-corrected) = 0.042] and accent recognition subscore was associated with the hinting task [*P* = 0.0039; *P*(FDR-corrected) = 0.03]. Tuning recognition was not associated with the social cognition tests. Next, we tested associations between the mean SCR during emotional music and the social cognition tests across all the groups and found no associations. Patients with auditory hedonic phenotypes (i.e. musicophilia, musical anhedonia, change in musical taste and sound/music aversion) performed similarly on social cognition tasks as FTD individuals without these phenotypes (all *P* > 0.05). The full overview of the associations of musicality subscores with social cognition subscores can be found in Supplementary Table 2. Next, we repeated the regression analyses in each group separately and found that in healthy subjects, better performance on the MEDT, the PROMS total, melody recognition and tuning recognition were all associated with better facial emotion recognition abilities (all *P* < 0.024). In the patients with bvFTD, better performance on the MEDT was associated with the better facial emotion recognition [*P* = 0.004; *P*(FDR-corrected) = 0.038] and executive functioning [*P* = 0.027; *P*(FDR-corrected) = 0.09], and better accent recognition was associated with the hinting task [*P* = 0.01; *P*(FDR-corrected) = 0.1]. In the analysis with Alzheimer’s disease patients only, we found no associations of musicality with the social cognition tests.

**Table 3:** Association between musicality and social cognition

	Ekman-60-faces test	Hinting task	RSMS	SNQ
bvFTD, Ad and HC				
MEDT—total	0.6681 (0.084)***	0.3651 (0.0997)***	0.125 (0.1317)	0.3246 (0.1226)*
PROMS—total	0.3657 (0.1148)**	0.3772 (0.1027)***	0.0382 (0.1465)	0.2247 (0.1307)
PROMS—Melody	0.3582 (0.1192)**	0.3141 (0.1099)**	0.0777 (0.1468)	0.2001 (0.1355)
PROMS—Tempo	0.3164 (0.1125)**	0.2913 (0.1041)**	−0.0274 (0.144)	0.1549 (0.1276)
PROMS—Accent	0.1644 (0.1143)	0.3004 (0.999)**	−0.0206 (0.1358)	0.1921 (0.1231)
PROMS—Tuning	0.2131 (0.1115)	0.201 (0.1021)	−0.0826 (0.1328)	0.1094 (0.1232)
bvFTD				
MEDT—Total	0.6663 (0.1893)**	0.4601 (0.2719)	0.3763 (0.2026)	0.0588 (0.2583)
PROMS—total	0.2205 (0.2817)	0.5804 (0.2771)	0.3994 (0.2403)	−0.0201 (0.2769)
PROMS—Melody	0.3364 (0.3107)	0.6259 (0.3171)	0.3948 (0.2626)	0.2617 (0.3029)
PROMS—Tempo	0.4769 (0.311)	0.6601 (0.3303)	0.5685 (0.3008)	0.3687 (0.4105)
PROMS—Accent	0.0975 (0.2551)	0.6493 (0.2183)*	0.2006 (0.2155)	0.0767 (0.2451)
PROMS—Tuning	0.0995 (0.2499)	0.0067 (0.282)	0.1824 (0.2235)	0.1904 (0.2349)
Ad				
MEDT—Total	0.2359 (0.1607)	0.0244 (0.194)	0.0044 (0.2268)	0.2074 (0.2439)
PROMS—Total	0.0433 (0.1576)	0.2022 (0.1707)	0.2381 (0.2599)	0.0059 (0.2296)
PROMS—Melody	0.0861 (0.1687)	0.3009 (0.1758)	0.1247 (0.2331)	0.0652 (0.2467)
PROMS—Tempo	0.0466 (0.1322)	0.0142 (0.1498)	0.3539 (0.2132)	0.0397 (0.1925)
PROMS—Accent	0.0041 (0.131)	0.124 (0.1444)	0.0057 (0.21)	0.0651 (0.1895)
PROMS—Tuning	0.2872 (0.1857)	0.293 (0.2128)	0.0824 (0.3151)	0.1945 (0.5069)
HC				
MEDT—Total	0.4352 (0.179)*	0.1409 (0.1689)	−0.3081) (0.3005)	0.3689 (0.3306)
PROMS—Total	0.2867 (0.1151)*	0.0442 (0.1043)	0.0898 (0.2224)	0.3065 (0.2097)
PROMS—Melody	0.3361 (0.0972)**	0.0343 (0.0968)	0.1634 (0.193)	0.3296 (0.1911)
PROMS—Tempo	0.0266 (0.1427)	−0.0103 (0.1154)	−0.2051 (0.2326)	0.1129 (0.2395)
PROMS—Accent	0.1129 (0.116)	0.0811 (0.0966)	0.0264 (0.2122)	0.3112 (0.1883)
PROMS—Tuning	0.2196 (0.0904)*	0.0817 (0.0798)	0.2021 (0.1691)	0.0598 (0.1711)

The beta-coefficients and standard errors of the associations of musicality tests with the social cognition tests are displayed. The scores of the musicality and social cognition tasks are *z*-transformed. All analyses were adjusted for age, sex, MMSE and musical training. MEDT, musical emotion discrimination task; PROMS, profile of music perception skills; RSMS, revised self-monitoring scale; SNQ, social norms questionnaire. Significant associations are displayed in bold. *P ≤ 0.05; **P ≤ 0.01; ***P ≤ 0.001.

### Neuroanatomical associations of musicality and social cognition

**Figure 4:**
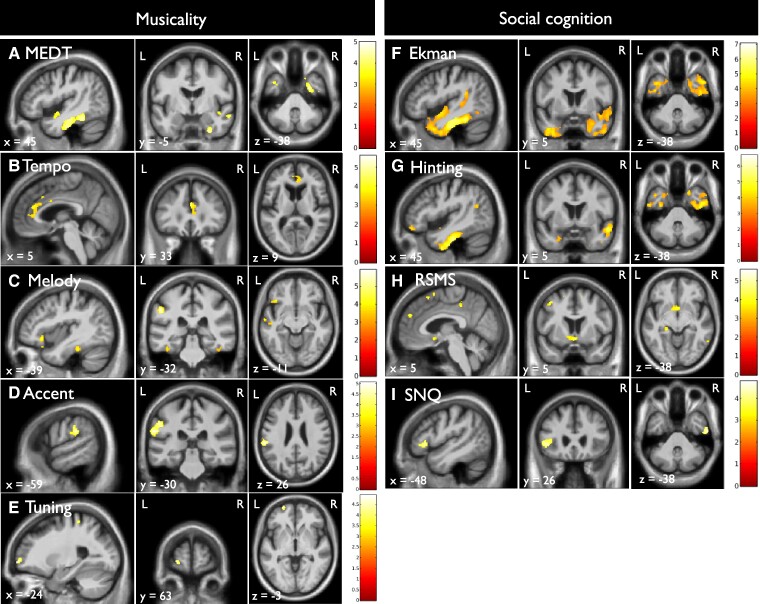
**Neuroanatomical associations of musicality and social cognition.** SPMs show regional grey matter volume positively associated with performance on (**A**) the MEDT, (**B**) tempo recognition, (**C**) melody recognition, (**D**) accent recognition, (**E**) tuning recognition, (**F**) Ekman-60-faces test, (**G**) hinting task, (**H**) RSMS and (**I**) SNQ. *T*-scores are coded on the colour bar. SPMs are thresholded at *P* < 0.001 uncorrected for multiple comparisons over the whole-brain volume and displayed on sections of a group mean T_1_-weighted MR brain template image in Montreal Neurological Institute standard space; age, sex, total intracranial volume, diagnosis and scanner type were included as covariates of no interest; significant clusters >50 voxels are shown; the left hemisphere is shown on the left of the axial and coronal sections. L, left side; R, right side; MEDT, musical emotion discrimination task; Ekman, Ekman-60-faces test; RSMS, revised self-monitoring scale; SNQ, social norms questionnaire.

**Figure 5:**
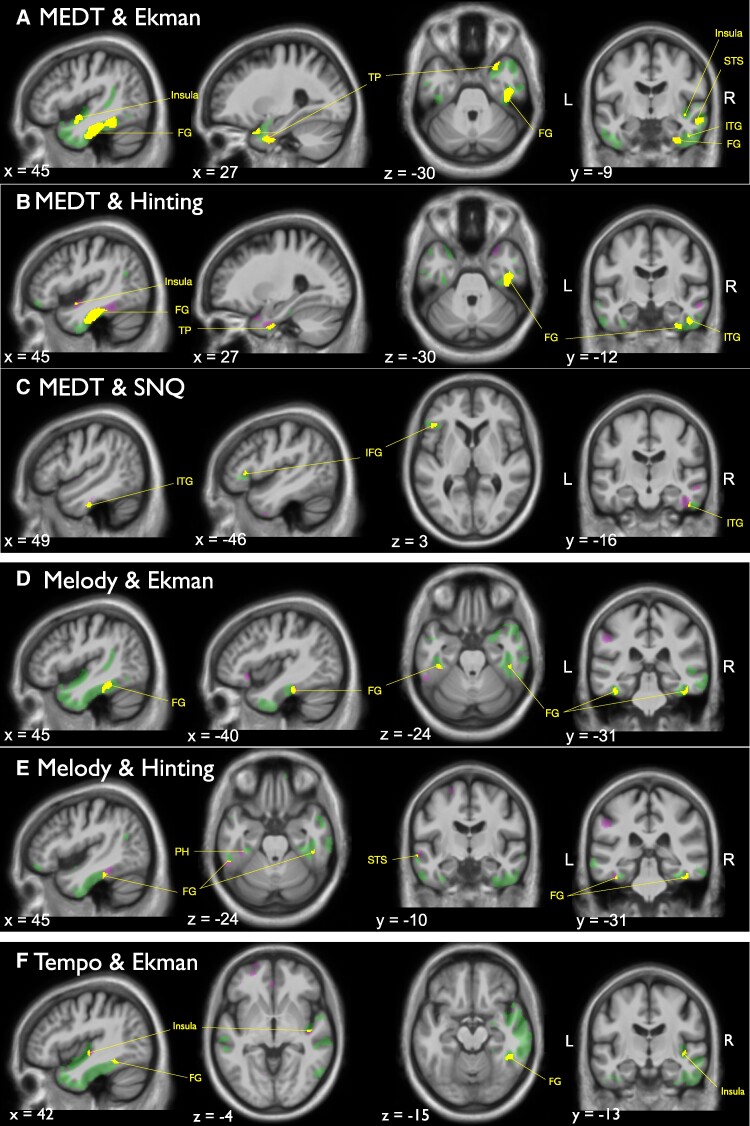
**Overlapping brain regions of the musicality and social cognition tests.** (**A**) Overlap between the MEDT and Ekman-60-faces test. (**B**) Overlap between the MEDT and hinting task. (**C**) Overlap between the MEDT and SNQ. (**D**) Overlap between the PROMS melody recognition subtest and the Ekman-60-faces test. (**E**) Overlap between the melody recognition subtest and the hinting task. (**F**) Overlap between the tempo recognition subtest and recognition and the Ekman-60-faces test. Overlapping brain regions of musicality and social cognition are shown in yellow, musicality tests are shown in magenta and social cognition tests are shown in green. SPMs are displayed on sections of a group mean T_1_-weighted MR brain template image in Montreal Neurological Institute standard space; the left hemisphere is shown on the left of the axial and coronal sections. R, right side; L, left side; FG, fusiform gyrus; TP, temporal pole; MTG, middle temporal gyrus; ITG, inferior temporal gyrus; IFG, inferior frontal gyrus; PH, parahippocampal gyrus; MEDT, musical emotion discrimination task; SNQ, social norms questionnaire; Ekman, Ekman-60-faces test.

Grey matter correlates of the musicality and social cognition tests can be found in Fig. 4 and Supplementary Table 3. First for musicality, we found that worse performance on the MEDT, melody recognition and tempo recognition were all associated with atrophy in the fusiform gyri. Additionally, worse MEDT scores were associated with predominantly right-sided anterior temporal atrophy extending caudally to the parahippocampal gyrus, insula and middle temporal gyrus. Worse tempo recognition scores were additionally associated with atrophy in frontal regions including the anterior cingulate cortex, orbitofrontal gyrus and the frontal pole. Worse melody, tempo and accent recognition were associated with atrophy of the left supramarginal gyrus. Worse tuning recognition was associated with atrophy of the frontal pole and the superior parietal lobule. Then, for social cognition, we found that lower facial emotion recognition and theory of mind scores were all associated with anterior temporal lobe atrophy including the temporal poles extending to the fusiform gyri, superior, middle and inferior temporal gyri. Lower RSMS scores were associated with atrophy of the ventromedial prefrontal cortex, the precuneus and basal forebrain. Lower scores on the SNQ were associated with atrophy of the left inferior frontal gyrus and right inferior temporal gyrus. Next, we investigated overlapping regions of the musicality and social cognition tests that showed significant associations in the linear regressions. The grey matter correlates of the overlapping brain regions of musicality and social cognition are displayed in Fig. 5. The MEDT showed overlap with both facial emotion recognition and the hinting task in anterior temporal regions including the temporal pole, the fusiform gyrus and the insula. The MEDT and SNQ showed overlap in the left inferior frontal gyrus, and right inferior temporal gyrus. Both melody and tempo recognition showed overlap with facial emotion recognition and theory of mind in the posterior fusiform gyri. Accent recognition and the hinting test showed no neuroanatomical overlap.

## Discussion

In this study, we investigated the relationship between alterations in musicality and social cognition, and their neuroanatomical correlates in a sample of patients with bvFTD, Alzheimer’s disease and controls. We found that musicality was impaired alongside social cognition in bvFTD, but not in Alzheimer’s disease. Multiple components of musicality were associated with emotion recognition and theory of mind. Furthermore, we found that worse performance on musicality tests (and social cognition) was associated with distinct brain regions including the temporal poles, fusiform gyri, and anterior cingulate cortex. These results suggest that musicality and social cognition share a neuroanatomical basis. Possibly, musicality tests may have potential diagnostic use as a probe for social cognition capacities.

The main finding of this study is that several components of musicality (music emotion recognition, tempo, accent and melody recognition) were associated with social cognition abilities (particularly facial emotion recognition and theory of mind), and with overlapping brain regions important for social cognition. It is important to consider and highlight the multidimensionality and interactions of such complex human traits, however. Multiple non-musical cognitive^34,35^ and demographic^36,37^ factors influence social cognition, and musicality is likely associated with other (non-social) capacities. Furthermore, general skills that are useful in musical context (e.g. predictive decoding of complex ‘rule-based’ temporal sequences) might be helpful in social context.^38^ However, musicality tests may be less influenced by cultural differences than social cognition.^39^ Taking these factors into account, our associations suggest that musicality could potentially probe components of social cognition, which aligns with previous research.^1-3,13^ However, there were no associations in the Alzheimer’s disease group, and music might therefore have a limited ability to probe social cognition in Alzheimer’s disease. The music emotion recognition test and tempo recognition test scores showed a good discriminatory ability (AUC >0.8) for detecting bvFTD, and future research could investigate the implementation of musicality tests, although implementation of musicality tests might be restricted to patients with normal hearing. Similar to a previous study on musicality,^40^ we found that musicality was associated with both acoustic (e.g. hinting task) and non-acoustic stimuli (e.g. facial emotion recognition test) suggesting common underlying mechanisms. Multiple musicality and social cognition tests overlapped neuroanatomically in the fusiform gyrus, which might reflect its role in the multisensory integration of emotional content,^41,42^ supporting both musical and social capacities. The superior temporal sulcus was also found in multiple musicality and social cognition tests, which might reflect its role in (visual and auditory) social perception^43,44^ as well as music perception.^45^ Furthermore, the association of various musicality tests (musical emotion recognition, melody recognition and musicality summed score) with facial emotion recognition remained significant in healthy controls, indicating potential generalizability of our results to the broader population. This association aligns with previous studies demonstrating enhanced emotion recognition in musicians.^46,47^ Overall, our findings emphasize the relationship between musicality and social cognition and underscore the need for further research to elucidate causality and the broader clinical implications of these connections.

Another finding of the current study is that music emotion recognition and tempo recognition were impaired in bvFTD but not in Alzheimer’s disease. Impairments of music emotion recognition in bvFTD have been described in previous studies,^7,9,14^ and we found similar anatomical associations in the temporal poles and fusiform gyri.^7,14^ The temporal poles are hubs of the semantic appraisal network,^15,48^ which is involved in emotion recognition.^48^ The overlapping clinical and anatomical findings of both musical- and facial emotions suggest that emotion recognition is not domain specific and might reflect general multidomain emotion processing deficits in bvFTD. Music emotion recognition was additionally associated with performance on the theory of mind task, which like previous findings^49^ overlapped anatomically in the right anterior temporal lobe. This might reflect the role of music in emotional inference,^1,2^ beyond what is required for general emotional decoding (e.g. emotion recognition). We further found worse tempo recognition in bvFTD compared to Alzheimer’s disease and controls. To our current knowledge, no previous studies have investigated tempo recognition in dementia. Tempo recognition was associated with facial emotion recognition and theory of mind, and with a cluster in the right anterior cingulate cortex, the right insula and fusiform gyrus, which are important brain regions for social cognition. The anterior cingulate cortex is a key hub of the salience network^50^ and closely linked to reward systems that facilitate social functions, such as making social prediction making,^51^ social learning^52^ and understanding affective states.^53^ It is also involved in rhythm processing,^54^ and a recent review proposes that the evolutionary advantages of music for social bonding might be largely driven by musical rhythm, by enhancing social predictability through reward systems.^55^ The anterior cingulate cortex might be an important brain region linking rhythm to social bonding, but future studies should further investigate this.

Our results suggest that impairments of musicality (acquired amusia) can develop as a clinical symptom in bvFTD.^56^ However, only two case reports have described acquired amusia as an early clinical manifestation of bvFTD.^57,58^ With the current study design, it is not possible to rule out the alternative hypothesis that premorbid musicality deficits predispose for bvFTD. Subjects with congenital amusia already have socio-emotional impairments,^40^ and frontotemporal anatomical anomalies,^59-62^ which might make them vulnerable for diseases where social cognition is affected. This idea fits with findings in primary progressive aphasia, where developmental learning difficulties are important neurodevelopmental risk factors.^63^ In a previous study, we found that earlier life musical training ameliorates socio-emotional functions in bvFTD,^64^ which might reflect the effects of musicality on socio-emotional resilience. Longitudinal and genetic studies in bvFTD and congenital amusia might give insights into potential developmental risk factors for bvFTD.

Although SCRs to emotional stimuli were lower in bvFTD compared to Alzheimer’s disease, in line with previous studies,^65,66^ we did not find an association of such responses with the social cognition test scores. This suggests that SCR has only limited capacity to reflect the socio-emotional changes in bvFTD. Furthermore, we found no differences in the performance on the social cognition tests in bvFTD patients with musicophilia compared to bvFTD patients without musicophilia, which is in contrast with a study by Fletcher *et al*.^13^ Possibly, this is due to the notion that our sample of bvFTD patients included only 6 musicophilia patients compared to 12 in the study, and so we were probably underpowered for reliable comparisons.

One potential limitation of the current study is that MRI data were available in a limited number (48/73; 65.8%) of subjects. Furthermore, multiple MRI scanners were used for the anatomical analysis, and although we adjusted for scanner-related effects, this may still have introduced noise in the data making it more difficult to find associations. Another potential limitation is the uncorrected threshold of *P* < 0.001 for the VBM analyses, as this can result in false positive results. However, we tried to limit this by only including larger clusters >50 voxels. The strength of the study is that we used validated musicality and social cognition metrics. Other strengths were that all participants were tested in a laboratory setting with a homogeneous and controlled test battery, and that we were able to combine clinical, biometric and neuroimaging data.

## Conclusion

We investigated musicality in dementia and found evidence for impairments of musicality in bvFTD compared to Alzheimer’s disease and healthy controls. Musicality was associated with emotion recognition and theory of mind abilities and with overlapping brain regions of social cognition. These results provide evidence into the biological correlates of music with a potential diagnostic utility of music as an indicator of social cognition capacities.

## Supplementary Material

fcae429_Supplementary_Data

## Data Availability

Data and stimuli that support the findings of this study are available from the corresponding author, upon reasonable request. The underlying code for this study is available via https://github.com/Jochumvanthooft/MELODIA_braincommunications.
