# Supplementary material for: Musicality and social cognition in dementia: clinical and anatomical associations
Source: Brain Commun. 2024 Dec 13;6(6):fcae429. doi: 10.1093/braincomms/fcae429 (PMC11642622; doi:10.1093/braincomms/fcae429)
Supplement: fcae429_Supplementary_Data [file fcae429_supplementary_data.pdf]

Supplementary material

Musicality and social cognition in dementia: clinical and anatomical associations

by J.J. van 't Hooft et al

Supplementary Table 1. Musical excerpts for music emotion induction (from Vuoskoski & Eerola 2011)

Excerpt	Emotion	Duration	Time
Oliver Twist	Happy	72s	01:32-02:09
Pride & Prejudice	Happy	56s	00:10-01:06
Batman Returns	Scary	46s	00:00-00:46
The Fifth Element	Scary	61s	00:00-01:01
The Portrait of a Lady	Sad	45s	00:00-00:23
Pride & Prejudice	Sad	50s	00:40-01:30

Supplementary Table 2. Association between musicality and social cognition subtests

	Ekman-60-faces test	Hinting task	RSMS	RSMS-EX	RSMS-SP	SNQ total	SNQ overadherence score	SNQ break score
**MEDT**								
MEDT – Total	**0.6681 (0.084)*****	**0.3651 (0.0997)*****	0.125 (0.1317)	0.1773 (0.1318)	0.0514 (0.1295)	**0.3246 (0.1226)***	**-0.3277 (0.1263)***	**-0.3079 (0.1231)***
**PROMS**								
PROMS – Total	**0.3657 (0.1148)****	**0.3772 (0.1027)*****	0.0382 (0.1465)	0.0565 (0.1478)	0.013 (0.1431)	0.2247 (0.1307)	-0.2091 (0.135)	-0.0896 (0.1402)
PROMS – Melody	**0.3582 (0.1192)****	**0.3141 (0.1099) ****	0.0777 (0.1468)	0.0629 (0.1484)	0.0868 (0.1431)	0.2001 (0.1355)	-0.1525 (0.1404)	-0.1208 (0.1441)
PROMS – Tempo	**0.3164 (0.1125)****	**0.2913 (0.1041)****	-0.0274 (0.144)	0.0109 (0.1454)	-0.0689 (0.1402)	0.1549 (0.1276)	-0.1587 (0.1312)	-0.0441 (-0.1587)
PROMS – Accent	0.1644 (0.1143)	**0.3004 (0.999)****	-0.0206 (0.1358)	0.0291 (0.1371)	-0.0761 (0.1312)	0.1921 (0.1231)	-0.0748 (0.1287)	-0.2033 (0.1293)
PROMS – Tuning	0.2131 (0.1115)	0.201 (0.1021)	-0.0826 (0.1328)	0.0617 (0.1344)	0.0984 (0.1294)	0.1094 (0.1232)	-0.2264 (0.1242)	0.1082 (0.1295)
Mean SCR - happy music	0.024 (0.1197)	0.0545 (0.0993)	0.1134 (0.1798)	0.1263 (0.1564)	0.0867 (0.2058)	0.3536 (0.1939)	-0.1832 (0.1644)	-0.3188 (0.2094)
Mean SCR - sad music	-0.01 (0.1128)	0.0376 (0.0934)	0.1001 (0.1666)	0.1093 (0.1452)	0.0791 (0.1905)	0.3661 (0.1795)	-0.1922 (0.1536)	-0.3269 (0.1951)
Mean SCR - fearful music	0.0022 (0.112)	0.0308 (0.0927)	0.0991 (0.1664)	0.1112 (0.1449)	0.0748 (0.1904)	0.339 (0.1805)	-0.177 (0.153)	-0.3038 (0.1953)
Musicophilia (FTD only)	0.3141 (0.556)	0.5983 (0.6762)	-0.5437 (0.4155)	0.4241 (0.5336)	-0.4241 (0.5336)	0.681 (0.5708)	-0.3981 (0.7069)	-0.5587 (0.8013)

The beta-coefficients and Standard Errors of the significant associations are displayed. The scores of the musicality and social cognition tasks are z-transformed. All analyses were adjusted for age, sex, MMSE and musical training. Significant associations are displayed in bold, * $P \leq 0.05$; **$P \leq 0.01$; ***$P \leq 0.001$.

Supplementary Table 3. Neuroanatomical associations of musicality and social cognition

Musicality test	Brain region	Side	Cluster size	Coordinates			*T* score
				x	y	z	
**MEDT**	Fusiform gyrus	R	1135	42	-44	-20	4.99
	Fusiform gyrus	R		39	-30	-22	4.77
	Fusiform gyrus	R		42	-21	-32	4.57
	Middle temporal gyrus	R	311	58	-8	-16	4.89
	Middle temporal gyrus	R		57	-18	-14	3.56
	Fusiform gyrus	R	338	28	-6	-40	4.66

	Temporal pole	L	200	-40	6	-36	3.93
	Inferior temporal gyrus	L		-44	2	-44	3.59
	Temporal pole	L		-34	2	-39	3.5
	Inferior frontal gyrus	L	93	-45	26	3	3.79
	Inferior frontal gyrus	L		-57	21	9	3.69
	Temporal pole	R	125	27	14	-30	3.75
	Temporal pole	R		20	14	-36	3.73
	Superior temporal gyrus	R	112	45	0	-10	3.72
	Superior temporal gyrus	R		50	-2	-3	3.42
	Posterior insula	R		40	-9	-9	3.37
**Tempo**	Calcarine gyrus	L	208	-8	-70	3	5.61
	Anterior cingulate gyrus	R	753	2	36	22	5.24
	Supplementary motor gyrus	R		6	21	38	4
	Anterior cingulate gyrus	R		10	38	4	3.89
	Supramarginal gyrus	L	109	-45	-33	44	4.94
	Superior frontal gyrus	R	59	20	45	44	4.84
	Middle frontal gyrus	L	68	-38	44	-2	4.74
	Superior frontal gyrus	L	118	-18	58	3	4
	Frontal pole	L		-22	64	-4	3.8
	Orbitofrontal gyrus	L	64	-22	52	-8	3.99
	Inferior temporal gyrus	R	72	46	-44	-14	3.81
	Anterior cingulate gyrus	L	55	-2	38	-6	3.56
	Superior temporal gyrus	R	60	44	-16	-4	3.52
**Melody**	Supramarginal gyrus	L	313	-51	-32	38	5.9
	Inferior frontal gyrus	L	178	-40	22	-8	4.39
	Temporal pole	L		-45	22	-16	3.65
	Orbitofrontal gyrus	L		-39	22	-22	3.63
	Middle temporal gyrus	L	67	-60	-12	-10	4.32
	Middle temporal gyrus	R	70	54	-58	-3	4.21
	Inferior frontal gyrus	L	74	-54	-45	-26	3.99
	Precentral gyrus	L	112	-21	-10	66	3.97
	Precentral gyrus	L		-22	-26	68	3.66
	Fusiform gyrus	L	90	-38	-34	-26	3.96
	Middle temporal gyrus	L	79	-52	-20	-15	3.87
	Fusiform gyrus	R	164	42	-40	-15	3.87
**Accent**	Supramarginal gyrus	L	671	-60	-30	26	5.33
	Supramarginal gyrus	L		-51	-28	38	4.84
	Postcentral gyrus	L		-44	-24	36	4.17
	Supramarginal gyrus	R	123	44	-42	50	5.25
	Ventral diencephalon	L	77	-16	-27	-6	4.05
**Tuning**	Frontal pole	L	120	-24	63	-3	4.9
	Superior parietal lobule	R	181	28	-45	45	4.81
	Postcentral gyrus	L	67	-20	-36	62	4.13
**Social cognition test**	**Brain region**	**Side**	**Cluster size**		**Coordinates**		***T* score**
				**x**	**y**	**z**	
**Ekman-60-faces**	Fusiform gyrus	R	10853	39	-28	-24	7.04
	Fusiform gyrus	R		44	-40	-22	6.36
	Inferior temporal gyrus	R		45	-16	-33	5.86
	Inferior frontal gyrus	L	1663	-40	-6	-44	5.02

	Temporal pole	L		-42	6	-38	4.87
	Inferior frontal gyrus	L		-45	-9	-34	4.87
	Fusiform gyrus	L	442	-39	-28	-26	4.94
	Inferior temporal gyrus	R	386	56	-62	-6	4.61
	Inferior temporal gyrus	R		46	-62	-10	3.66
	Inferior temporal gyrus	R		64	-54	-10	3.62
	Middle temporal gyrus	L	205	-58	-36	0	4.53
	Superior temporal sulcus	L	154	-56	-27	10	4.33
	Lingual gyrus	R	66	24	-38	-12	4.18
	Planum temporale	R	52	63	-20	15	4.1
	Transverse temporal gyrus	R		52	-20	10	3.37
	Superior temporal gyrus	L	132	-57	-24	-3	4.09
	Middle temporal gyrus	L	129	-57	0	-20	4.06
	Planum polare	L		-45	-2	-22	4
**Hinting task**	Inferior frontal gyrus	L	157	-54	-40	-21	6.45
	Inferior frontal gyrus	R	2554	46	-18	-33	5.57
	Fusiform gyrus	R		33	-10	-45	5.24
	Inferior frontal gyrus	R		44	-12	-44	5.09
	Superior temporal gyrus	R	795	54	6	-18	5.21
	Temporal pole	R		50	14	-10	3.93
	Middle temporal gyrus	R		51	0	-28	3.52
	Orbitofrontal gyrus	R	190	42	51	-16	4.66
	Middle temporal gyrus	R	801	66	-26	-4	4.64
	Middle temporal gyrus	R		63	-24	-22	4.14
	Middle temporal gyrus	R		58	-24	-15	4.04
	Inferior temporal gyrus	L	183	-56	-10	-34	4.45
	Inferior temporal gyrus	L		-51	-16	-30	4.03
	Middle temporal gyrus	L	326	-63	-30	-12	4.39
	Middle temporal gyrus	L		-62	-36	-2	4.01
	Middle temporal gyrus	L	144	-62	-52	-2	4.34
	Temporal pole	R	183	21	12	-38	4.27
	Temporal pole	R		22	12	-46	4.25
	Middle temporal gyrus	L	334	-60	-3	-18	4.15
	Middle temporal gyrus	L		-60	-20	-12	3.8
	Planum polare	L		-46	-2	-20	3.77
	Fusiform gyrus	L	377	-34	-12	-36	4.1
	Fusiform gyrus	L		-32	-26	-30	4.09
	Fusiform gyrus	L		-36	-32	-26	3.74
	Middle temporal gyrus	R	242	63	-48	4	4.1
	Middle temporal gyrus	R		63	-48	-4	3.83
	Angular gyrus	R	103	44	-57	21	4.04
	Temporal pole	L	86	-50	12	-32	4.03
	Temporal pole	L	172	-30	16	-30	4.02
	Orbitofrontal gyrus	R	91	14	60	-21	3.93
**RSMS**	Precuneus	L	454	-2	-54	46	5.57
	Precuneus	L		-2	-63	45	4.8
	Precuneus	L		-10	-58	46	4.15
	Angular gyrus	L	215	-30	-69	51	5.28
	Angular gyrus	L		-26	-74	44	4.66
	Angular gyrus	L		-34	-74	44	3.58
	Middle medial frontal gyrus	L	444	-26	18	50	5.17
	Superior medial frontal gyrus	L		-21	27	51	4.76

	Superior medial frontal gyrus	L		-16	20	52	4.74
	Middle temporal gyrus	L	495	-52	-38	-6	4.9
	Superior temporal gyrus	L		-54	-48	9	3.94
	Superior temporal gyrus	L		-58	-27	0	3.77
	Superior parietal lobule	R	181	14	-46	63	4.75
	Superior parietal lobule	R		22	-51	68	4.06
	Postcentral gyrus	R		21	-38	68	3.45
	Superior temporal gyrus	R	393	60	-36	4	4.73
	Middle temporal gyrus	R		52	-40	-3	4.22
	Superior temporal gyrus	R		46	-33	8	3.72
	Supplementary motor gyrus	R	158	10	16	57	4.5
	Supplementary motor gyrus	R		3	9	63	4.06
	Superior medial frontal gyrus	R		12	2	62	3.78
	Nucleus accumbens	R	210	2	9	-10	4.5
	Putamen	L		-10	6	-8	3.7
	Inferior temporal gyrus	R	88	51	-48	-20	4.4
	Parahippocampal gyrus	L	106	-21	-32	-10	4.29
	Hippocampus	L		-22	-24	-18	3.57
	Middle temporal gyrus	R	101	60	-57	-6	4.27
	Middle frontal gyrus	L	78	-38	9	50	4.24
	Middle frontal gyrus	L		-45	4	40	3.47
	Superior medial frontal gyrus	R	94	4	50	28	4.23
	Superior medial frontal gyrus	R		12	44	28	3.68
	Posterior cingulate gyrus	R	69	6	-33	44	4.03
	Precuneus	R		6	-36	52	3.68
	Cerebellum	L	198	-8	-60	-54	3.92
	Cerebellum	L		-14	-57	-63	3.47
	Planum temporale	L	61	-63	-24	9	3.72
**SNQ**	Inferior temporal gyrus	R	304	58	-10	-39	4.77
	Inferior temporal gyrus	R		51	-16	-36	3.94
	Inferior temporal gyrus	R		57	-20	-32	3.65
	Inferior frontal gyrus	L	371	-50	27	2	4.34
	Inferior frontal gyrus	L		-46	34	-2	3.66
	Superior frontal gyrus	L	70	-4	14	69	3.92
	Middle frontal gyrus	R	57	38	28	34	3.78
	Middle frontal gyrus	R		40	36	33	3.53
	Middle frontal gyrus	R	102	24	45	18	3.65
	Middle frontal gyrus	R		33	44	15	3.63

The regions shown were all significantly associated with the musicality and social cognition tests thresholded at $P < 0.001$ uncorrected for multiple comparisons over the whole brain volume. All significant clusters >50 voxels are shown. Peak (local maximum) coordinates are in Montreal Neurological Institute (MNI) standard stereotactic space.
